# Morphologic and molecular classification of lung neuroendocrine neoplasms

**DOI:** 10.1007/s00428-020-03015-z

**Published:** 2021-01-21

**Authors:** Jasna Metovic, Marco Barella, Fabrizio Bianchi, Paul Hofman, Veronique Hofman, Myriam Remmelink, Izidor Kern, Lina Carvalho, Linda Pattini, Angelica Sonzogni, Giulia Veronesi, Sergio Harari, Fabien Forest, Mauro Papotti, Giuseppe Pelosi

**Affiliations:** 1 Department of Oncology, University of Turin, Turin, Italy; 2 Inter-Hospital Pathology Division, Servizio Interaziendale di Anatomia Patologica, IRCCS MultiMedica, Via Gaudenzio Fantoli 16/15, 20138 Milan, Italy; 3 Cancer Biomarker Unit, Fondazione IRCCS Casa Sollievo della Sofferenza, San Giovanni Rotondo, Italy; 4 Laboratory of Clinical and Experimental Pathology, FHU OncoAge, Nice Hospital, Biobank BB-0033-00025, IRCAN, Inserm U1081 CNRS 7284, University Côte d’Azur, Nice, France; 5 Department of Pathology, Erasme Hospital, Université Libre de Bruxelles (ULB), Route de Lennik 808, 1070 Brussels, Belgium; 6 Cytology and Pathology Laboratory, University Clinic of Respiratory and Allergic Diseases, Golnik, Slovenia; 7 Department of Pathological Anatomy, Coimbra Hospital and University Center, Coimbra, Portugal; 8 Department of Electronics, Information and Bioengineering, Politecnico di Milano, Milan, Italy; 9 Department of Pathology and Laboratory Medicine, IRCCS Istituto Nazionale dei Tumori, Milan, Italy; 10 Division of Thoracic Surgery, San Raffaele Scientific Institute – IRCCS, Milan, Italy; 11 School of Medicine, Vita-Salute San Raffaele University, Milan, Italy; 12 Department of Medical Sciences and Community Health, University of Milan, Milan, Italy; 13 Division of Pneumology, San Giuseppe Hospital, IRCCS MultiMedica, Milan, Italy; 14 Department of Pathology, University Hospital Center (CHU), North Hospital, Saint-Étienne, France; 15 Department of Oncology and Hemato-Oncology, University of Milan, Milan, Italy

**Keywords:** Neuroendocrine, Lung, Carcinoma, Carcinoid, Typical, Atypical, Large cell, Small cell, Classification, Stem cell, Progression, Differentiation, Gene, Molecular, Signature

## Abstract

Neuroendocrine neoplasms (NENs) of the lung encompass neuroendocrine tumors (NETs) composed of typical (TC) and atypical (AC) carcinoids and full-fledged carcinomas (NECs) inclusive of large cell neuroendocrine carcinoma (LCNEC) and small cell carcinoma (SCLC). NETs and NECs are thought to represent distinct and separate lesions with neither molecular overlap nor common developmental continuum. Two perspectives were addressed regarding the morphologic and molecular classification of lung NENs: (i) a supervised approach by browsing the traditional classification, the relevant gene alterations, and their clinical implications; and (ii) an unsupervised approach, by reappraising neoplasms according to risk factors and natural history of disease to construct an interpretation model relied on biological data. We herein emphasize lights and shadows of the current classification of lung NENs and provide an alternative outlook on these tumors focused on what we currently know about the biological determinants and the natural history of disease.

## Introduction

**Fig. 1:**
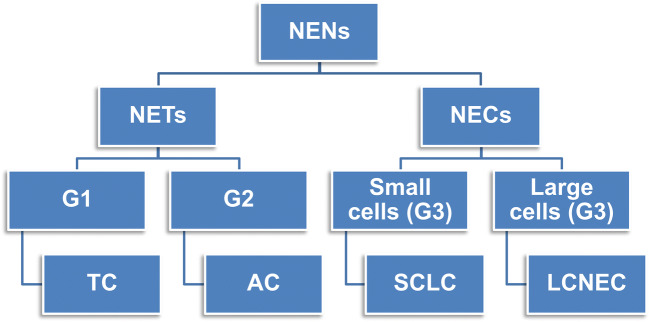
Composition of lung neuroendocrine neoplasms. A binary subdivision into neuroendocrine tumors (NETs) and neuroendocrine carcinoma (NECs) is preliminary to the identification of typical carcinoid (TC) as G1 tumor, atypical carcinoid (AC) as G2 tumor, and NEC with small cells (namely small cell lung carcinoma, SCLC) and large cells (namely large cell neuroendocrine carcinoma, LCNEC), both of them making tautologically G3—high-grade neoplasms

Lung neuroendocrine neoplasms (NENs) encompass four histologic subtypes whose terminology and defining criteria have been endorsed over the last three classifications by the World Health Organization (WHO). This classification will remain fundamentally unchanged in the forthcoming 5th edition of the WHO Blue Book on lung tumors. Accordingly, lung NENs include typical carcinoid (TC), atypical carcinoid (AC), large cell neuroendocrine carcinoma (LCNEC), and small cell lung carcinoma (SCLC) (Fig. 1). Diagnostic criteria include mitotic count per 2 mm^2^ and necrosis alongside a wide constellation of cellular and architectural features fulfilling NE morphology, while immunohistochemical (IHC) markers are applied to reveal NE differentiation especially in high-grade SCLC and LCNEC (Table 1). The most important separation is between well differentiated tumors of low (i.e., TC as G1) to intermediate (i.e., AC as G2) grade (henceforth, NETs) and poorly differentiated full-fledged NE carcinomas (henceforth, NECs), which are all grouped together as high-grade neoplasms (tautologically G3) with small cell (SCLC) and/or large cell (LCNEC) appearance and no remarkable differences in survival between them [62] (Fig. 1). The morphologic approach to classification complies with a three-tier spectrum of clinical outcomes, survival rates, and therapy options, essentially because defining criteria are effective enough to highlight low-to-intermediate grade while failing to a large extent in the behavioral distinction of NECs. Such a dichotomy between NETs and NECs has strict biological grounds, inasmuch as NETs are not precursors to NECs and therapy options are closely dictated upon morphology-based tumor grading [75]. Molecular investigations have indeed supported the notion that NETs make up distinct and separate tumor entities as opposed to NECs, arguing against a causative relationship between NETs and NECs (Table 2).

**Table 1:** Main pathologic and immunohistochemistry features of lung neuroendocrine neoplasms. *TC*, typical carcinoid; *AC*, atypical carcinoid; *LCNEC*, large cell neuroendocrine carcinoma; *SCLC*, small cell lung carcinoma; *NE*, neuroendocrine; *IHC*, immunohistochemistry; *CgA*, chromogranin A; *Syn*, synaptophysin; *INSM1*, insulinoma-associated protein 1; *RB*, retinoblastoma gene product; *p53*, p53 protein; *SSTR*, somatostatin receptors

Parameter	TC	AC	LCNEC	SCLC
Mitotic count per 2 mm^2^	0–1	2–10	> 10	> 10
Necrosis	No	Punctate	Extensive	Geographic
Cell size	Variable (variants)	Variable (variants)	Large	Small (< 3 resting lymphocytes)
Nuclear chromatin	Finely granular (salt and pepper texture)	Finely granular (salt and pepper texture)	Coarse to vesicular	Evenly distributed and finely granular
Nucleoli	Occasional, small	Common, small	Common, large	Inconspicuous, small
Cytoplasm	Variable	Variable	Abundant	Scarce
Pattern growth	Organoid, trabecular, nesting	Organoid, trabecular, nesting	Organoid, trabecular, palisading	Solid to diffuse, sheet-like, roughly trabecular
NE morphology	Yes	Yes	Yes	Yes
Combined variants	No	No	Yes	Yes
Ki-67*	< 10%	10–25%	25–80%	70–100%
IHC marker				
CgA	+++	++	+	±
Syn	+++	+++	+++	± to +++**
INSM1	+++	+++	+++	+++
RB	+++	+++	±	–
p53	–	±	+/++	+++
SSTR	+++	+++	+/++	±

*Ki-67 is quantified according to the percentage of nuclear-labeled cells over 2000 elements or per 2 mm^2^ in areas of the highest staining (hot spots). IHC results are shown semiquantitatively on a scale from negative (−) to 3 +: ± corresponds to immunoreactivity in up to 10% neoplastic cells, 1 + to 11–25%, 2 + to 26–50%, and 3 + to over 50%

**About 10% of SCLC cases belonging to the so-called variant subtype shows negative to faint expression of synaptophysin and other neuroendocrine markers, including INSM1

**Table 2:** Main molecular alterations across the spectrum of lung neuroendocrine neoplasms. Common molecular alterations of pulmonary low- and high-grade neuroendocrine tumors. *TC*, typical carcinoid; *AC*, atypical carcinoid; *LCNEC*, large cell neuroendocrine carcinoma; *SCLC*, small cell lung carcinoma. *MEN1*, menin 1 gene; *TP53*, tumor protein p53 gene; *RB1*, retinoblastoma 1 gene; *EGFR*, epidermal growth factor receptor gene; *PIK3CA*, phosphatidylinositol-4,5-bisphosphate 3-kinase catalytic subunit alpha gene; *PTEN*, phosphate and tensin homolog gene; *NOTCH 1*, notch 1 gene; *KMT2A*, lysine methyltransferase 2A; *MYCL1*, v-myc avian myelocytomatosis viral oncogene lung carcinoma–derived homolog gene; *FGFR1*, fibroblast growth factor receptor 1 gene. For details on the prevalence of the single-gene alterations in each category of lung NENs, see the text. In this synoptic table, 3+ stands for frequent, 2+ for occasional, and 1+ for uncommon

Symbol	Mapping to	Type of molecular alteration	TC	AC	LCNEC	SCLC
MEN1	11q13.1	Mutation	+++	++	Absent/rate	Absent/rate
TP53	17p13.1	Mutation loss	Absent/rate	Absent/rate	++	+++
RB1	13q14.2	Mutation loss	Absent/rate	Absent/rate	++	+++
EGFR	7q11.2	Mutation	+	+	+	Absent/rare
PIK3CA	3q26.32	Mutation	+	+	+	Absent/rare
PTEN	10q23.31	Mutation	Absent/rare	Absent/rare	+	+
NOTCH1	9q34.3	Mutation	Absent/rare	Absent/rare	++	++
KMT2A	11q23.3	Mutation	+	+	+	++
MYC	8q24.21	Amplification fusion	Absent/rare	Absent/rare	+	++
MYCN	2p24.3					
MYCL1	1q34.2					
FGFR1	8p11.23	Mutation Amplification	Absent/rare	Absent/rare	+	+

Herein, we aimed to review lights and shadows of lung NEN classification by accounting for their clinicopathologic features, molecular traits, and natural history of disease.

## Material and methods

An accurate survey of papers related to tumor classification and intratumor heterogeneity (not a systematic review nor meta-analysis) was conducted until the end of July 2020. A list of key questions regarding lung NENs was generated concerning diagnosis, prognosis, classification, tumor subtypes, carcinoid, small cell carcinoma, large cell neuroendocrine carcinoma, variant subtype, prediction, immunohistochemistry, clinical implications, genetic/epigenetic changes, and development of biomolecular models. Only articles dealing with the 2015 WHO classification were accounted for to assure comparability of both pathology criteria and clinical outcomes. The research was limited to available English literature in PubMed®, with only full-papers being considered along with some smaller studies or even case reports, if appropriate. Morphologic and molecular classification of lung NENs was addressed under two different perspectives: (i) a supervised one, by browsing the traditional classification through its own specific gene alterations and clinical implications, and (ii) an unsupervised one, by reappraising these neoplasms according to risk factors, molecular assembly, and developmental mechanisms.

### A supervised approach: a classification based on morphology with clinical claims

#### Histologic classification

**Fig. 2:**
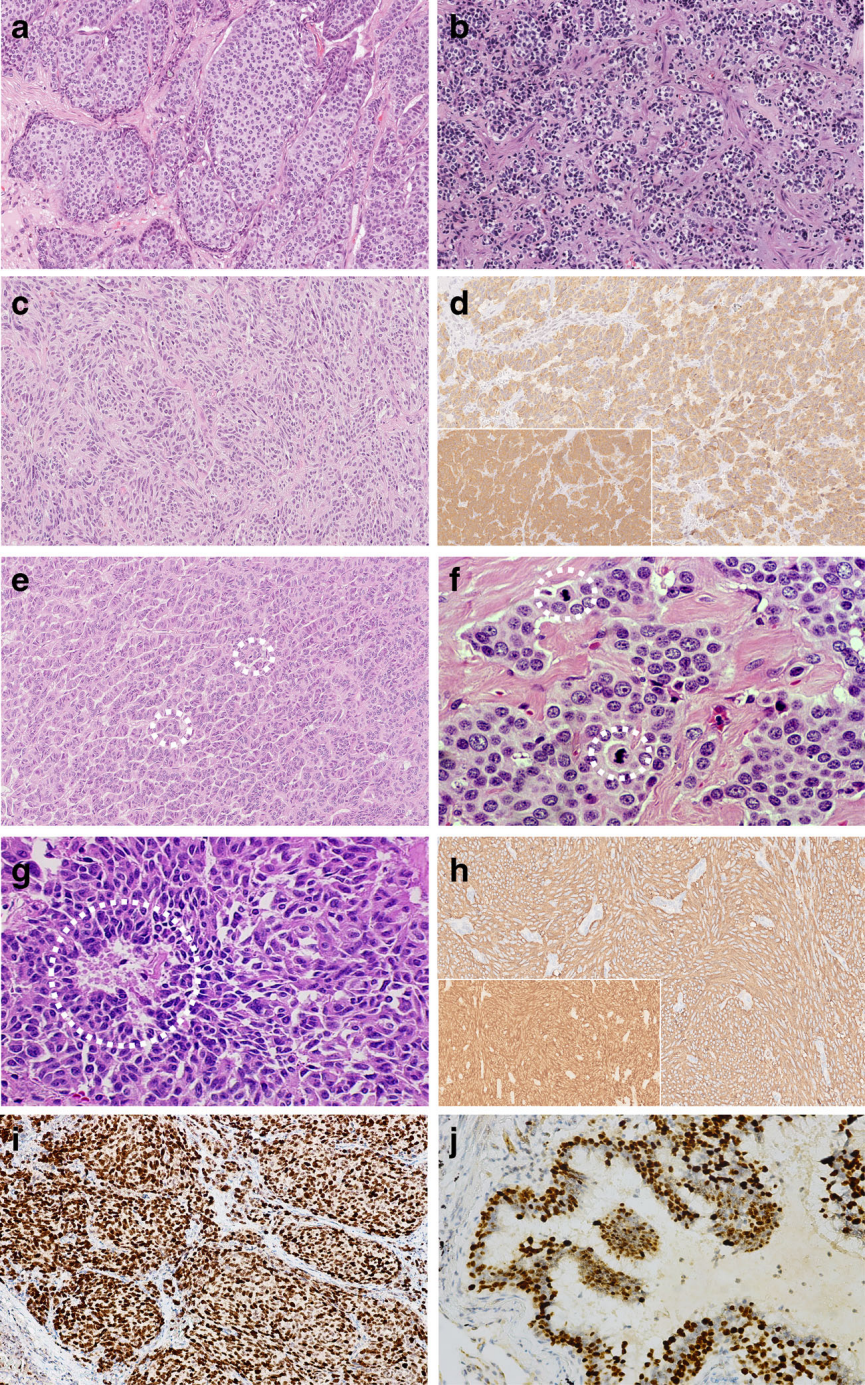
Histomorphologic and immunohistochemical features of lung carcinoids. Typical carcinoid shows a variety of histological patterns, including organoid to trabecular (**a**), lobular (**b**), or spindle cells (**c**), but always with < 2 mitoses per 2 mm^2^ and absent necrosis. Immunohistochemistry for neuroendocrine markers documents diffuse and intense positivity for chromogranin A (**d**) and synaptophysin (**d**, inset). This atypical carcinoid featuring a trabecular architecture exhibits at least two mitoses (**e**, dotted white circle), which are also easily recognizable in another similarly patterned case (**f**, dotted white circle). Atypical carcinoid may exhibit punctate necrosis, in this case against a background of a solid-appearing tumor (**g**, dotted white circle). Immunohistochemistry for neuroendocrine markers in atypical carcinoid documents reactivity for synaptophysin (**h**), chromogranin A (**h**, inset) and INSM1 (**i**), all consistent with neuroendocrine morphology. Of note, INSM1 is also expressed in normal and hyperplastic neuroendocrine cells, as well as in tumorlets (not shown in the picture) (**j**)

**Fig. 3:**
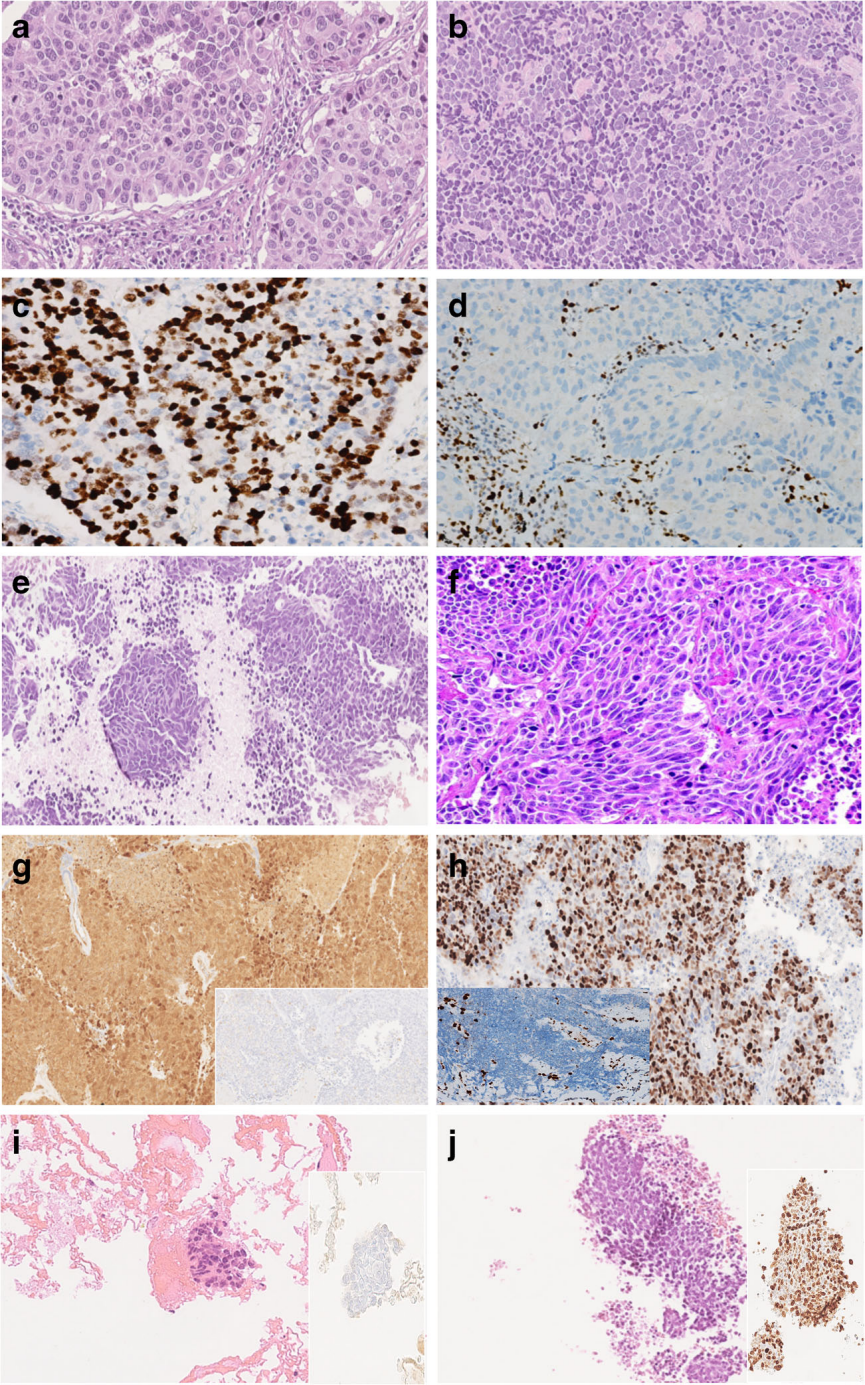
Histomorphologic and immunohistochemical features of lung neuroendocrine carcinomas**.** LCNEC features organoid aggregates with peripheral palisading and plentiful mitoses (**a**), but even SCLC-like appearance (**b**). LCNEC is usually positive for INSM1 (**c**), while retinoblastoma nuclear decoration is often missing (**d**). SCLC is composed of small-sized tumor cells with prominent nuclear molding, inconspicuous nucleoli, and abundant necrosis (**e**), but spindling of neoplastic cells may be on record (**f**). Synaptophysin is largely retained in this case (**g**), along with INSM1 labeling (**h**), while chromogranin A is usually reduced to negative (**g**, inset), just like does retinoblastoma that is consistently unreactive (**h**, inset). Differential diagnosis between carcinoids (**i**) and SCLC (**j**) may be demanding upon morphology in cytology samples, but Ki-67 staining helps to operate such a separation confidently showing low expression in carcinoid (**i**, inset) and high expression in SCLC (**j** inset). Note that distinction of typical and atypical carcinoids rests on mitotic count and/or punctate necrosis, while neuroendocrine carcinomas are especially distinguished according to cell size appreciation.

Lung NENs make up about 20% of all lung malignancies, with a large prevalence of NECs (LCNEC and SCLC) over NETs (TC and AC). These four histologic subtypes are distinguished upon mitotic count per 2 mm^2^, necrosis assessment, and cytologic detail appreciation (i.e., relative cytoplasm amount, nuclear chromatin pattern, nucleolar appearance, nucleus-cytoplasm ratio) (Table 1). NE morphology in lung NETs shows an extreme variety of features, with multiple patterning (e.g., insular, lobular, trabecular, follicular, and solid) and/or cellular variants (polygonal, spindle, oncocytic, mucinous, melanin-laden, etc.) within the same tumors (Table 1 and Fig. 2). Likewise, the same wide array of morphologic features can be documented in NECs, whether LCNEC or SCLC, where cellular details (cell size and nuclear chromatin patterning) serve as discriminants to distinguish LCNEC (large cells with coarser chromatin and prominent nuclei) from SCLC (small cells with finely dispersed chromatin and inconspicuous nucleoli) (Table 1 and Fig. 3). NECs have no upper limits in mitotic count, generally demonstrating around 50–60 mitoses in LCNEC and 70–80 mitoses in SCLC [75], with variable necrosis amount accounting for prognosis deterioration [55, 61].

The concurrence of combined variants is a nearly exclusive prerogative of approximately 25–30% NECs [75]. In SCLC, combined variants are defined by the association with any other subtype of non-small cell carcinoma (NSCC) (LCNEC, adenocarcinoma, squamous cell carcinoma, sarcomatoid carcinoma, or large cell carcinoma), while combined LCNEC shows adjuncts exclusively of other NSCC subtypes [42, 75]. Just the association of SCLC and LCNEC requires a threshold of 10% for either tumor component to be present because of the continuum for cell size and nuclear changes [75]. Combined variants of NETs (mostly AC) with adenocarcinoma or squamous carcinoma are de facto anecdotal findings [28, 36, 39] and those with NECs are undescribed [32, 75].

Classification of lung NENs is applicable as a whole to surgical specimens only, whereas cytology/small biopsy samples need more simplified terminology, especially in the event of crush artifacts [3, 11, 41]. TC and AC are grouped together as carcinoids, and LCNEC is only suggested as possible option of diagnosis.

#### The integration of immunohistochemistry

As a part of morphologic NE differentiation, lung NENs show consistent IHC expression of pan-NE markers, such as chromogranin A, synaptophysin, or insulinoma-associated protein 1 (INSM1), the latter being a newly released marker of NE differentiation in the lung [15, 16, 63], effective even in cytology/biopsy samples [1] (Table 1). In NSCC lacking NE morphology, the current guidelines discourage performing NE markers. A minority of SCLC (about 10–15%) shows low/absent levels of NE markers, realizing the so-called variant subtype, which molecularly straddle morphologically undifferentiated NSCC and LCNEC [65, 69, 83].

The IHC characterization is a defining criterion in LCNEC to rule out NSCC, but is recommended in SCLC to improve its differential diagnosis towards close histologic mimickers, such as poorly differentiated squamous cell carcinoma and basaloid carcinoma, small round cell tumors, NUT carcinoma, or hematologic malignancies [74]. No diagnostic role is currently supported in lung NENs for Ki-67 labeling index because of the significant overlap between tumor categories. However, it can be helpful in small biopsies with crush artifacts or cytology samples in order to avoid overdiagnosing carcinoids as SCLC [3, 41].

In a wider context of differential diagnosis, IHC markers may help to exclude histologic mimickers, especially when facing with NECs [47, 81]. Briefly, (i) positive expression of a couple of NE immunomarkers (expectedly chromogranin A and synaptophysin ± INSM1) in the lung serves to put NE differentiation into the proper context of morphologic features, sample types (cytology, biopsy, resection), and extent of positive reaction [81]. Of note, INSM1 reactivity is consistently found not only across the entire spectrum of lung NENs but also even in normal and reactive NE cells as documented in Fig. 2j at variance with Achaete-Scute family BHLH transcription factor 1 (*ASCL1*) which is mostly expressed by NECs. CD56 should be considered an indicator of SCLC [27] rather than a marker of NE differentiation due to lack of specificity; (ii) p40 [43, 44] and/or high molecular weight cytokeratins [70] exclude, if negative, poorly differentiated squamous cell carcinoma and basaloid carcinoma; (iii) TTF1 rules out, if positive, the NE-low variant subtype of SCLC [23, 83]; (iv) cytokeratins are useful to highlight either epithelial differentiation or staining pattern, i.e., dot-like vs. cytoplasmic diffuse (lung carcinoids may be proven cytokeratin-negative especially in peripheral tumors, but separation from paraganglioma is clinically negligible); (v) retinoblastoma identifies, if positive, carcinoids and some NECs straddling LCNEC and SCLC and facing with the variant subtypes of SCLC [69]; (vi) p53 abnormal expression, either diffuse staining or absence in all tumor cells, is commonly shared by subtypes of NECs but can also be found in carcinoids, albeit rarely [67]; (vii) lastly, demonstration of NUT protein identifies NUT carcinoma, regardless of the remaining IHC patterning [81].

Beyond diagnosis, several IHC biomarkers can be useful in the emerging clinical setting of targeted therapies and immuno-oncology setting of lung NENs. They include somatostatin receptors in NETs [60]; delta-like canonical Notch Ligand 3 (DLL3), an inhibitory NOTCH ligand, in SCLC [38]; mammalian target of rapamycin (mTOR) in NETs and LCNEC [59, 82]; and programmed death-ligand 1 (PD-L1) [76] or thymidylate synthase (TS) [24] in NECs.

#### Clinical correlations

A direct inference of the histological classification occurs in clinical behavior, according to which TC are low-grade (G1) malignant tumors with long survival and good prognosis even in the event of distant metastases, AC intermediate grade (G2) malignant tumors with significantly increased rates of distant metastases and considerably worse prognosis [6, 22, 62], and NEC full-fledged high-grade carcinomas (tautologically G3) with the highest likelihood of lymph node and visceral metastases and dismal prognosis in spite of therapy efforts [9, 25, 35] (Table 3).

**Table 3:** Main clinical features of lung neuroendocrine neoplasms. *TC*, typical carcinoid; *AC*, atypical carcinoid; *LCNEC*, large cell neuroendocrine carcinoma; *SCLC*, small cell lung carcinoma: *M*, males; *F*, females; *LD*, limited disease; *ED*, extensive disease; *I*, stage I of disease; *IV*, stage IV of disease

Parameter	TC	AC	LCNEC	SCLC
Age* (mean)	45	55–60	65	75
Sex	M < F	M = F	M > F	M > F
Smoking	No	Variable	Yes	Yes
Paraneoplastic syndromes	10–15%	40–48%	90%	90–100%
Visceral metastases	3–5%	20–30%	50%	75%
5-year survival	92–98%	40–70%	13–17%	15–20% in LD < 1% in ED
10-year survival	87–90% I → 96% IV → 59%	42–64% I → 88% IV → 18%	None	None
Survival over 10 years	83%	58%	None	None

*Expressed in years (mean value)

Paraneoplastic syndromes can be observed in either NETs or NECs, whether endocrinological or non-endocrinological. SCLC account for the most frequent lung cancer associated with paraneoplastic syndromes (up to 20% of instances), with either ectopic hormone production (hyponatremia, Cushing syndrome) or autoimmune-mediated destruction upon onconeural neoantigen expression (paraneoplastic encephalomyelitis or dermatomyositis with poorer outcome and Lambert-Eaton myasthenic syndrome with more prolonged clinical course). In LCNEC, paraneoplastic syndromes are quite uncommon, with single records of ectopic adrenocorticotropic hormone syndrome (EACTH) and Lambert-Eaton syndrome or cancer-associated retinopathy. In NETs, TCs are the major reservoir of paraneoplastic syndromes (5%) as compared to AC (< 2%), mostly of endocrinological type (EACTH [29], GH-related acromegaly, histamine-related atypical carcinoid syndrome), but even of non-endocrinological type (giant cell arteritis, rheumatic polymyalgia, or polymyositis). Regarding demography, NECs by far prevail in elderly male smokers, while NETs, particularly TC, slightly exceed in younger female non-smokers (Table 3). Regarding treatment, NETs are primarily managed by surgery, deserving multimodal approaches to metastatic cases (especially AC) [4, 6, 21, 22], whereas NECs are mostly treated by (neo)adjuvant chemoradiotherapy [9, 21, 25, 26], a deserving surgery to very early-stage lesions (5% or less of patients) [31]. Options of targeted therapies and/or immunoncology scenarios, which are being increasingly explored in both NETs and NECs, also require accurate subtyping of lung NEN patients.

#### Molecular pathology

Pulmonary NENs encompass a substantially heterogeneous spectrum of molecular alterations, whose profiling could aid in the clinical management of patients by means of tailored treatments.

Regarding low-grade arm, Fernandez-Cuesta et al. performed whole-exome sequencing (WES) of 69 pulmonary carcinoids identifying alterations of chromatin remodeling genes, namely, menin 1 (*MEN1*), AT-rich interaction domain 1A (*ARID1A*), and eukaryotic translation initiation factor 1A X-linked (*EIF1AX*), as significantly mutated genes along with low-mutation burden (a mean somatic mutation rate of 0.4 mutations per megabase (Mb) of sequenced DNA) [12]. These molecular findings were confirmed by subsequent studies, which also argued against genetic separation of carcinoids likely due to the existence of common precursors [30, 67]. In a case series examined by Swarts et al. [73], 7/55 (13%) of pulmonary carcinoids harbored *MEN1* mutations associated with reduced mRNA expression and poor prognosis. Also, in mutation-absent tumors, low *MEN1* gene expression was correlated with an adverse disease outcome [73]. In keeping with the abovementioned data, WES analysis of 14 pulmonary carcinoids (10 TC and 4 AC) confirmed a low mean somatic mutation rate of 0.3 per Mb [34]. Moreover, the number of somatic alterations was associated with increasing proliferation activity with both Ki67 index (*p* < 0.01) and mitotic count (*p* < 0.01). On the other hand, Mucin 6 (*MUC6*) and spectrin alpha erythrocytic 1 (*SPTA1*) were recurrently mutated at a frequency of 21% (3/14) and 14% (2/14), respectively. Of note, pathway analysis of the mutated genes revealed enrichment of genes involved in mitogen-activated protein kinase (MAPK) signaling, regulation of the actin cytoskeleton and focal adhesion, and transforming growth factor (TGF)-β signaling [34]. Despite small sample size, 307 genes were found to be differentially expressed to a significant degree in 5 pulmonary carcinoids with favorable prognosis as compared with 5 cases with an adverse clinical course. Within all carcinoids as well as atypical carcinoids, RET proto-oncogene (*RET*) upregulation and orthopedia homeobox gene (*OTP*) and CD44 molecule (Indian blood group) downregulation were significantly associated with low 20-year survival of patients [71, 72]. Moreover, chromosomal instability, in terms of increased frequency and extent of chromosomal alterations, was found as a common event in atypical and metastasized carcinoids [79].

The literature data indicate that the genetic profile of pulmonary carcinoids is considerably different from that of high-grade NECs, which demonstrate an overall high-tumor mutation burden (> 7 per megabase pairs) [19, 64]. The mutation frequency increases with the tumor’s higher grade or biological malignancy as heralded in other lung cancer subtypes [46]. In this regard, in a paper by Vollbrecht et al., the SMAD family member 4 (*SMAD4*) mutation was found in TC subtype, while KIT proto-oncogene receptor tyrosine kinase (*c-KIT*), phosphatase and tensin homolog (*PTEN*), HNF1 homeobox A (*HNF1A*), and smoothened frizzled class receptor (*SMO*) were altered in AC [78]. On the other hand, Janus kinase 3 (*JAK3*), NRAS proto-oncogene (*NRAS*), RB transcriptional corepressor 1 (*RB1*), and Von Hippel-Lindau tumor suppressor (*VHL1*) were exclusively identified in SCLC, whereas the fibroblast growth factor receptor 2 (*FGFR2*) mutation was detected in LCNEC only [78]. Of note, *JAK3* mutations were also found in LCNEC of the thymus, an organ where the classification criteria of NENs are the same as in the lung, which secondarily developed from preexisting AC [10]. In experimental models, inhibition of *NOTCH* gene signaling induces lung progenitor cells to form pulmonary neuroendocrine cells, from which tumors resembling early-stage SCLC grew in immunodeficient mice via *RB* and *TP53* simultaneous blockage and transcriptome enrichment with cell cycle–related RNAs [7].

LCNEC make up the widest spectrum of molecular heterogeneity among lung NENs, with some of them being classified as SCLC-like LCNEC accounting for about 40%, NSCLC-like LCNEC accounting for about 50% and carcinoid-like LCNEC accounting for about 5% on the basis of different sets of altered genes [57, 58]. SCLC-like LCNEC share molecular alterations with SCLC and show *RB1*, tumor protein P53 (*TP53*), CREB binding protein (*CREBBP*), E1A binding protein P300 (*EP300*), and lysine methyltransferase 2A (*KMT2A*) gene mutations alongside V-myc myelocytomatosis viral oncogene homolog 1 (*MYCL1*) and *FGFR1* amplifications. NSCLC-like LCNEC exhibit cyclin-dependent kinase inhibitor 2A (*CDKN2A*) deletion, transcription termination factor 1 (*TTF1*) amplifications, and Kelch-like ECH-associated protein 1 (*KEAP1*) and serine/threonine kinase 11 (*STK11*) mutations as observed in non-NE tumors. Finally, carcinoid-like LCNEC bear *MEN1* mutations [8, 57]. Interestingly, there is also a molecular link between NE-low and *RB1*-wild type LCNEC and variant subtypes of SCLC [65] that exhibit either low levels of NE differentiation or larger cell morphology, thus challenging once again LCNEC as a unitary neoplastic entity [69].

In a more recent study by Simbolo et al. [68] that investigated a case series consisting of AC and LCNEC, three transcriptional clusters were identified. *Cluster 1* comprised 20 LCNECs and one AC harboring concurrent inactivation of *TP53* and *RB1*, in the absence of *MEN1* mutations and RB1 nuclear decoration. *Cluster 2* included 14 AC and eight LCNEC showing intermediate features: inactivation of *TP53*, 40.9%; *MEN1*, 22.7%; and *RB1*, 18.2%. *Cluster 3* comprehended 20 AC and four LCNEC lacking *RB1* alterations and having frequent *MEN1* (37.5%) and *TP53* mutations (16.7%). Moreover, menin nuclear immunostaining was lost in 75% of cases. Notably, patients in cluster C1 had a shorter cancer-specific survival than did patients in C2 or C3, thus reflecting the importance of gene signature for tumor behavior as already stated [2, 50, 51, 68].

Zhou et al. [84] performed capture-based ultra-deep targeted sequencing on tumor samples of LCNEC, large cell carcinoma (LCC), and SCLC, revealing a molecular signature consisting of RUNX family transcription factor 1 (*RUNX1*), Erb-B2 receptor tyrosine kinase 4 (*ERBB4*), breast cancer 1 (*BRCA1*), and EPH receptor A3 (*EPHA3*), distinctively mutated in LCNEC. A majority (60%) of LCNEC patients harbored copy number variations (CNVs). Notably, NFKB inhibitor alpha (*NFKBIA*) amplification was shared between LCNEC and LCC, while AKT serine/threonine kinase 2 (*AKT2*) amplification was private to LCNEC and SCLC. There were no common CNVs shared among the three investigated subtypes. Furthermore, genetic alterations in the PI3K/AKT/mTOR pathway were enriched in all three subtypes, thus indicating converging effector mechanisms on carcinogenesis.

Uccella et al., casting 6 TC, 4 AC, 11 LCNEC, and 8 SCLC, demonstrated a significantly higher expression of ribonuclease T2 (RNASET2), hypoxia-inducible factor 1 subunit alpha (*HIF-1α*), and its target carbonic anhydrase IX (*CA IX*) in NECs than NETs [77]. Moreover, in vitro data showed that an overexpression of RNASET2 is consequence of the activation of HIF-1α. The authors suggested that RNASET2 increased expression may contribute to phenotypic alterations of NECs, such as the high apoptotic rate and the extensive necrosis [77].

In a study by George et al., two molecular subgroups of LCNECs were identified by means of comprehensive genomic and transcriptomic analyses [20], namely, “type I LCNEC” with biallelic *TP53* and serine/threonine kinase 11/Kelch-like ECH-associated protein 1 (*STK11/KEAP1*) alterations (37%), and “type II LCNECs” enriched for biallelic inactivation of *TP53* and *RB1* (42%). LCNECs form distinct transcriptional subgroups with closest similarity to SCLC. While type I LCNECs and SCLCs exhibited a neuroendocrine profile with *ASCL1*-high/delta-like canonical Notch Ligand 3 (*DLL3*) high/Notch Receptor 1 (*NOTCH*) low, type II LCNECs bore *TP53* and *RB1* alterations and differed from most SCLC tumors for reduced neuroendocrine markers, a pattern of ASCL1-low/DLL3-low/NOTCH-high and upregulation of immune-related pathways [20].

SCLC typically has one of the highest mutation rates in cancers, bearing biallelic inactivation of *TP53* and *RB1* as a hallmark [19, 40, 52], quite uncommon in TC and AC [5, 67]. Also, chromatin remodeling genes (*CREBBP*, *EP300*, *KMT2A*) are commonly found altered [12, 19, 40, 52]. Of note, in addition to mutual biallelic *RB1* and *TP53* alterations, *NOTCH* gene inactivation with simultaneous *ASCL1* and canonical *WNT* signaling engagement is the basis of pulmonary and extra-pulmonary small cell carcinoma developing as secondary tumors from preexisting non-NE carcinomas, either spontaneous or induced by therapy [33, 49–51]. A novel molecular stratification of SCLC tumors has recently been proposed by Rudin et al. [65] by integrating experimental data with clinical material and based on the relative abundance of genes encoding four key transcriptional factors, namely, *ASCL1*, neuronal differentiation 1 (*NEUROD1*), POU class 2 homeobox 3 (*POU2F3*), and yes1-associated transcriptional regulator (*YAP1*), as ASCL1-high (SCLC-A), NEUROD1-high (SCLC-N), YAP1-high (SCLC-Y), and POU2F3-high (SCLC-P) subtypes. SCLC-Y and SCLC-P merged with NE-low variant subtypes of this tumor, which share similarities to either LCNEC or NSCC [69]. We have incorporated some molecular characteristics of both low- and high-grade lung NENs into Table 2.

### An unsupervised approach: the issue of intratumor heterogeneity of classification

#### How to account for heterogeneity

The classification of lung NENs considers every different tumor category as a monolithic entity independent of each other rather than a combination of biologically diverse lesions, with major differences likely depending on natural history of disease (preclinical and clinical phases). It is tempting to hypothesize that the shorter the preclinical phase, the greater the clinical aggressiveness and the shorter the clinical course, with more severe gene alterations being responsible for undifferentiated tumors with cancer stem cell properties to arise. These tumors are likely to show resistance to therapy [51]. Conversely, the longer the preclinical phase, the lower the clinical aggressiveness and the more prolonged the clinical course, in the premise that over time accumulation of diverse gene alterations would cause variably differentiated tumors with progenitor cell properties to arise. These tumors are likely to be more amenable to therapy [50, 51]. In this scenario, tumor fate, clinical outcome, and susceptibility to therapies of lung NENs might be largely pre-determined at the onset and/or modeled over time by diverse molecular signatures, which in turn are the mirror of different risk factors acting persistently on lung tissue microenvironment [33, 50, 51]. Of note, while both the cells of origin and the driver genetic alterations are likely to play a role in shaping ultimate lung cancer phenotypes, the relationship between ancestors and cancer subtypes is not necessarily one-to-one, inasmuch as different sets of genetic/epigenetic alterations could result in variably differentiated and/or reprogrammed cancer cells [13, 14, 53, 54, 56, 66, 80]. This challenging interaction between ancestors and molecular alterations, in turn continuously under the influence of risk factors, gives rise to the important phenomena of intertumoral and intratumoral heterogeneity, subtype transdifferentiation, cell pliancy, and latency of lung tumors, which represent the pathologic and molecular bases of clinical outcome and potential targets of therapy [14, 50, 51, 56, 66]. All these issues, however, are only partially accounted for by the current classification, because defining criteria are not precise enough to intercept the great variety of neoplasms at the level of an individual patient’s cancer and thus to account for the natural history of tumors as the major determinant of their fate [51]. AC and LCNEC show the least reproducible diagnostic criteria and thus the broadest range of biological properties (prognosis, expression profiles, carcinogenesis paths, sensitivity to therapies), which is much less remarkable in either TC (well differentiated cells with negligible proliferation activity and no necrosis) or SCLC (undifferentiated small cells resembling stem cells with huge proliferation activity and geographic necrosis) (Table 1, 2, and 3). This intratumor heterogeneity of lung NEN classification can be unraveled by focusing on tumor grading, interobserver variability, expression profiles and single-gene alterations, survival analysis, omics studies, and pathogenesis models [45, 48, 50, 51].

#### Modeling the pathogenesis of lung neuroendocrine neoplasms

**Fig. 4.**
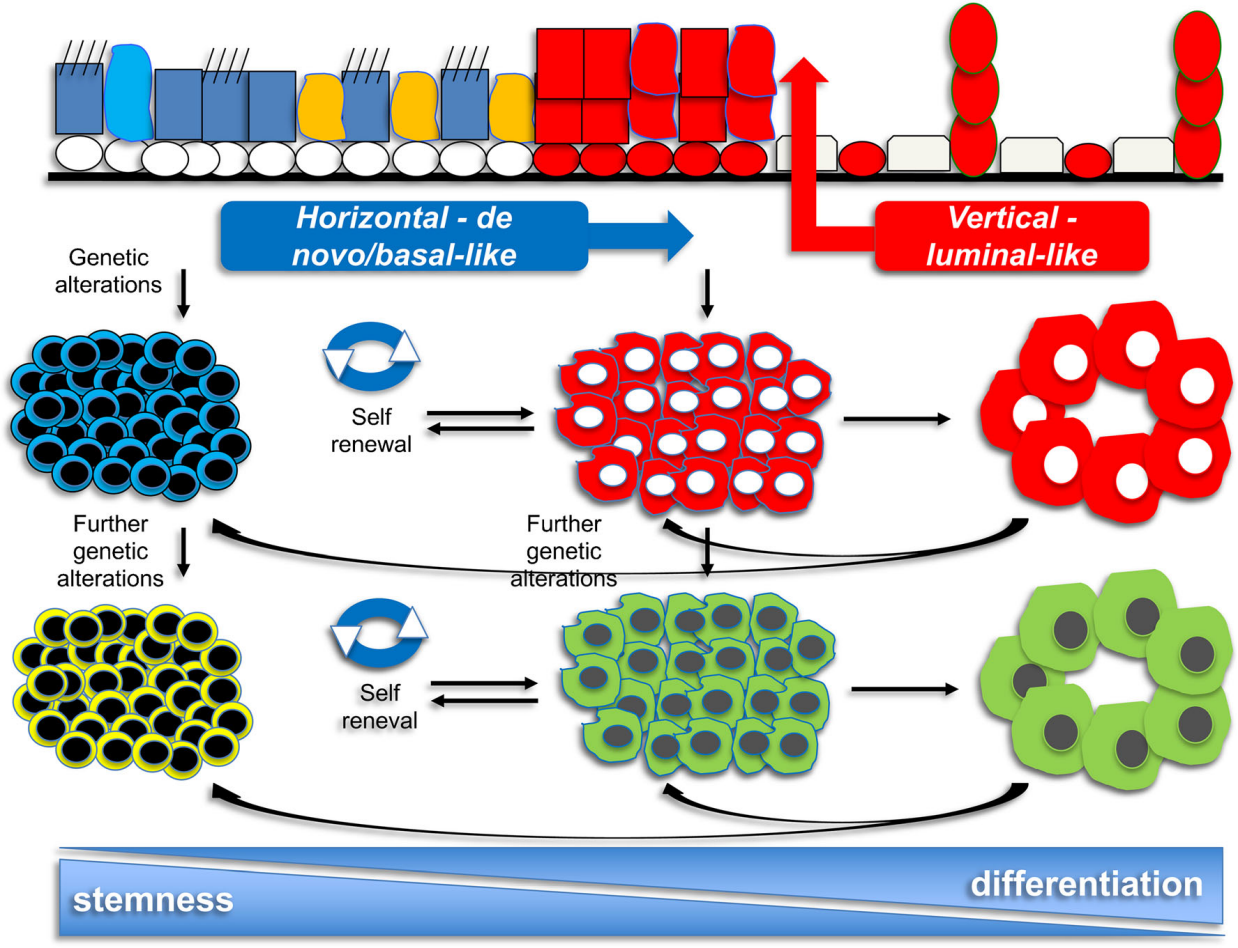
Development model of lung neuroendocrine neoplasms. Mechanisms of horizontal or de novo/basal-like propagation from cancer stem cells early blocked-in differentiation give rise to primary early-aggressive high-grade tumors (PEA-HGNETs/NECs). They are characterized by undifferentiated cells resembling typical SCLC featuring diffuse pattern of growth. Mechanisms of vertical- or luminal-like propagation from progenitor cells with an intermediate block of differentiation cause more differentiated lesions to develop from pre-existing neuroendocrine tumors or non-small cell carcinoma (NET-like DS-HGNET/NEC and NSCC-like DS-HGNET/NEC, respectively). They are characterized by better differentiated cells featuring a variety of histologic appearances along with organoid patterns of growth. Lastly, there are indolent NETs (I-NETs) composed of fully differentiated cells (in red and green on the right of picture), which usually do not progress further over time but can contribute to DS-HGNETs/NECs by re/deprogramming events. PEA-HGNET/NEC, primary early-aggressive high-grade neuroendocrine tumor/carcinoma; DS-HGNET/NEC, differentiating secondary high-grade neuroendocrine tumor/carcinoma; I-NET, indolent NET

The homeostasis of epithelial cells is maintained by an asymmetrical self-renewal of stem cells putatively aligned along basal layer of stratified epithelia or lineage compartments (horizontal or de novo/basal-like propagation) and differentiation of progenitor elements committed to specific cell lineages (vertical or luminal-like evolution). In this regard, tumor population could variably comprise cancer stem cells (CSC), adult-differentiated elements genetically reprogrammed during tumorigenesis (cell pliancy), progenitor elements derived from CSCs, deprogrammed cells, and differentiating cells, thereby realizing a heterogeneous assembly whose relative composition might result from different gene alterations and hence developmental mechanisms [14, 51, 66]. Of note, retinoblastoma (RB1) and TP53 genes suppress self-renewal, whereas Notch marks stem cells and initiates deprogramming and transit amplification [37]. Accordingly, this reciprocal proportion of transformed cells could reflect different genetic/epigenetic mechanisms of tumorigenesis and/or microenvironment stimuli, which could be related to and/or maintained by risk factors, either exogenous or endogenous, acting during the natural history of disease. Therefore, we can schematically identify three different types of neoplastic lesions. Each one of them is likely to derive from different mechanisms [51]. This interpretation model is inspired by an unsupervised approach to genetic signatures, which are likely to shape the final morphologic appearance of tumor cells [50] (Fig. 4).

- An early maturation block caused by crucial genetic events (e.g., biallelic inactivation of RB1 and TP53, NOTCH silencing) would promote horizontal or de novo/basal-like expansion and/or re/deprogramming of differentiated and/or progenitor cells. These mechanisms result in tumors enriched in neoplastic stem cells which are undifferentiated, with high biological malignancy and short preclinical phase. While dysplastic/in situ lesions composed of undifferentiated tumor cells have been experimentally documented in mouse models [17, 18], in humans, the smoking-damaged normal epithelium associated with SCLC is genetically scrambled by similar molecular alterations. These tumors, which can be further modulated by additional genetic alterations giving rise to similar lesions still dominated by CSCs in pure or combined forms, might derive from primordial adult cells out of a neuroendocrine niche even capable of multidivergent evolution [33, 66]. Therefore, they would represent primary or early-aggressive high-grade NETs (PEA-HGNETs/NECs) as a function of their short natural history as reflected by very short clinical course [51]. Transversions C > A are usually present due to their close relationship with smoking. Classical SCLC usually feature diffuse growth of undifferentiated cells (sometimes mimicking hematologic malignancies) [18], with possible multidivergent differentiation but still in a background of diffuse pattern of growth. These tumors are usually diagnosed on biopsy/cytology samples because of advanced disease at presentation, show low mutational heterogeneity between primaries and metastases, and are resistant to therapy with dismal prognosis [51]. These tumors show very high Ki-67 labeling index (usually 60–100%), with homogeneous distribution of stained cells inside tumors. PEA-HGNETs/NECs could account for 70–75% of lung NETs and about 13% of all lung cancers [51].
- An intermediate maturation block caused by a variety of gene alterations, such as TP53 → RB1 mono/biallelic inactivation, NOTCH alteration, KRAS/LKB1/MEN1 mutation, MYC/MYCL/TERT/SDHA/RICTOR amplification, or epithelial-mesenchymal transition, would promote vertical or luminal-like mechanisms and/or re/deprogramming of differentiated cells giving rise to tumors enriched in progenitor cells. They show a greater spectrum of morphologic differentiation and less biological aggressiveness. Transversions C > A are still present due to their association with smoking. These tumors can be further modeled by additional genetic alterations and are enriched by progenitor and better differentiated cells. They would be likely to derive from neuroendocrine and non-neuroendocrine precursors such as carcinoids or their ancestors or NSCC. Therefore, they would represent differentiating or secondary high-grade NETs (DS-HGNETs/NECs) as a function of their longer natural history [33, 50, 51]. Carcinoids with elevated indices of proliferation, LCNEC resembling NSCC or carcinoid and SCLC are usually seen with organoid patterns of growth even in combined variants [33, 50, 51]. Other features of these DS-HGNETs/NECs are lower stage at presentation, diagnosis on resection specimens, better prognosis, higher mutational heterogeneity between primaries and related metastases [80], and greater curability rates. These tumors present with a variable Ki-67 labeling index (ranging from 10 to 60%), typically with a heterogeneous distribution inside tumors. DS-HGNETs/NECs could account for 20–25% of lung NENs and about 6% of all lung cancers [51].
- A late maturation block caused by genetic/epigenetic alterations without genetic segregation would promote vertical luminal-like mechanisms giving rise to tumors enriched in differentiated cells, with low-grade dysplastic lesions (e.g., diffuse idiopathic neuroendocrine cell hyperplasia, DIPNECH), long preclinical phase and best prognosis. Transversions C > A are quite uncommon, but these smoking-linked tumors could be at higher risk of progression to DS-HGNETs/NECs [50]. Tumors would derive from mature NE/preinvasive lesions (e.g., DIPNECH) for genetic re/deprogramming and represent indolent NETs (I-NETs) with long-term preclinical phase [51]. These tumors mostly feature TC or low proliferating AC (e.g., ≤ 3 mitoses per 2 mm^2^), with organoid pattern of growth, diagnosis performed on resection specimens, low stage at presentation with favorable clinical outcome, and best curability upon surgery. I-NETs exhibit a low Ki-67 labeling index (≤ 10%), typically featuring homogeneous distribution inside tumors. They could account for 5% of lung NETs and about 1% of all lung cancers [51].

Of note, Ki-67 labeling index could be a major forerunner to this classification, with a homogeneous intratumor distribution in PEA-HGNETs/NECs and I-NETs, and an uneven distribution in DS-HGNETs/NECs due to coexistence of closely intermingling low and high proliferating tumor areas. Integration of the Ki-67 labeling index with the WHO classification could help to better stratify patients for different therapy options [48, 49].

## Conclusions

The WHO classification of lung NENs is an indispensable tool in clinical practice. Separation between NETs and NECs is fundamental to provide accurate and adequate management of patients considering significant differences in molecular landscapes, survival expectancy, and therapy options. However, while privileging an unsupervised approach upon molecular signatures, it is possible to reappraise lung NENs according to differences in risk factors and pathogenesis models where intratumor heterogeneity of Ki-67 labeling index could play an important role.
